# Acetylcholinesterase promotes apoptosis in insect neurons

**DOI:** 10.1007/s10495-020-01630-4

**Published:** 2020-08-05

**Authors:** Debbra Y. Knorr, Nadine S. Georges, Stephanie Pauls, Ralf Heinrich

**Affiliations:** Department of Cellular Neurobiology, Johann-Friedrich-Blumenbach-Institute for Zoology and Anthropology, Georg-August-University Göttingen, Göttingen, Germany

**Keywords:** Acetylcholinesterase, Non-synaptic, Apoptosis, Hypoxia, Insect, *Ace-1*

## Abstract

Apoptosis plays a major role in development, tissue renewal and the progression of degenerative diseases. Studies on various types of mammalian cells reported a pro-apoptotic function of acetylcholinesterase (AChE), particularly in the formation of the apoptosome and the degradation of nuclear DNA. While three AChE splice variants are present in mammals, invertebrates typically express two *ache* genes that code for a synaptically located protein and a protein with non-synaptic functions respectively. In order to investigate a potential contribution of AChE to apoptosis in insects, we selected the migratory locust *Locusta migratoria.* We established primary neuronal cultures of locust brains and characterized apoptosis progression in vitro. Dying neurons displayed typical characteristics of apoptosis, including caspase-activation, nuclear condensation and DNA fragmentation visualized by TUNEL staining. Addition of the AChE inhibitors neostigmine and territrem B reduced apoptotic cell death under normal culture conditions. Moreover, both inhibitors completely suppressed hypoxia-induced neuronal cell death. Exposure of live animals to severe hypoxia moderately increased the expression of *ace-1* in locust brains in vivo. Our results indicate a previously unreported role of AChE in insect apoptosis that parallels the pro-apoptotic role in mammalian cells. This similarity adds to the list of apoptotic mechanisms shared by mammals and insects, supporting the hypothesized existence of an ancient, complex apoptosis regulatory network present in common ancestors of vertebrates and insects.

**Electronic supplementary material:**

The online version of this article (10.1007/s10495-020-01630-4) contains supplementary material, which is available to authorized users.

## Introduction

Apoptosis describes highly regulated processes that lead to death and elimination of individual cells with little or no negative impact on the surrounding tissue. It contributes to the development of structured organs, to the renewal of adult tissue by regular turnover of cells and to the removal of compromised or malfunctioning cells (Reviewed: [1–4]). Morphologically, apoptosis is characterized by loss of cellular volume, condensation of nuclear chromatin due to DNA fragmentation, plasma membrane blebbing and formation of apoptotic bodies. While these morphological features are partly shared by other types of programmed cell death within and outside the animal phylum, apoptosis in its strict sense is executed by metazoan-specific cysteine proteases termed caspases [5–7]. Recent studies indicated that most components of the vertebrate-typical apoptosis regulatory network were already present in an early ancestor of all metazoans (Reviewed [5, 8]). Consequently, the well investigated comparatively simple apoptotic networks of *Caenorhabditis elegans* and *Drosophila melanogaster* resulted from a secondary reduction of ancient complexity. Whether this simplification is specific for theses model organisms or whether it is a general feature of nematodes, insects and their last common ecdysozoan ancestors is not clear at the present state.

**Fig. 1:**
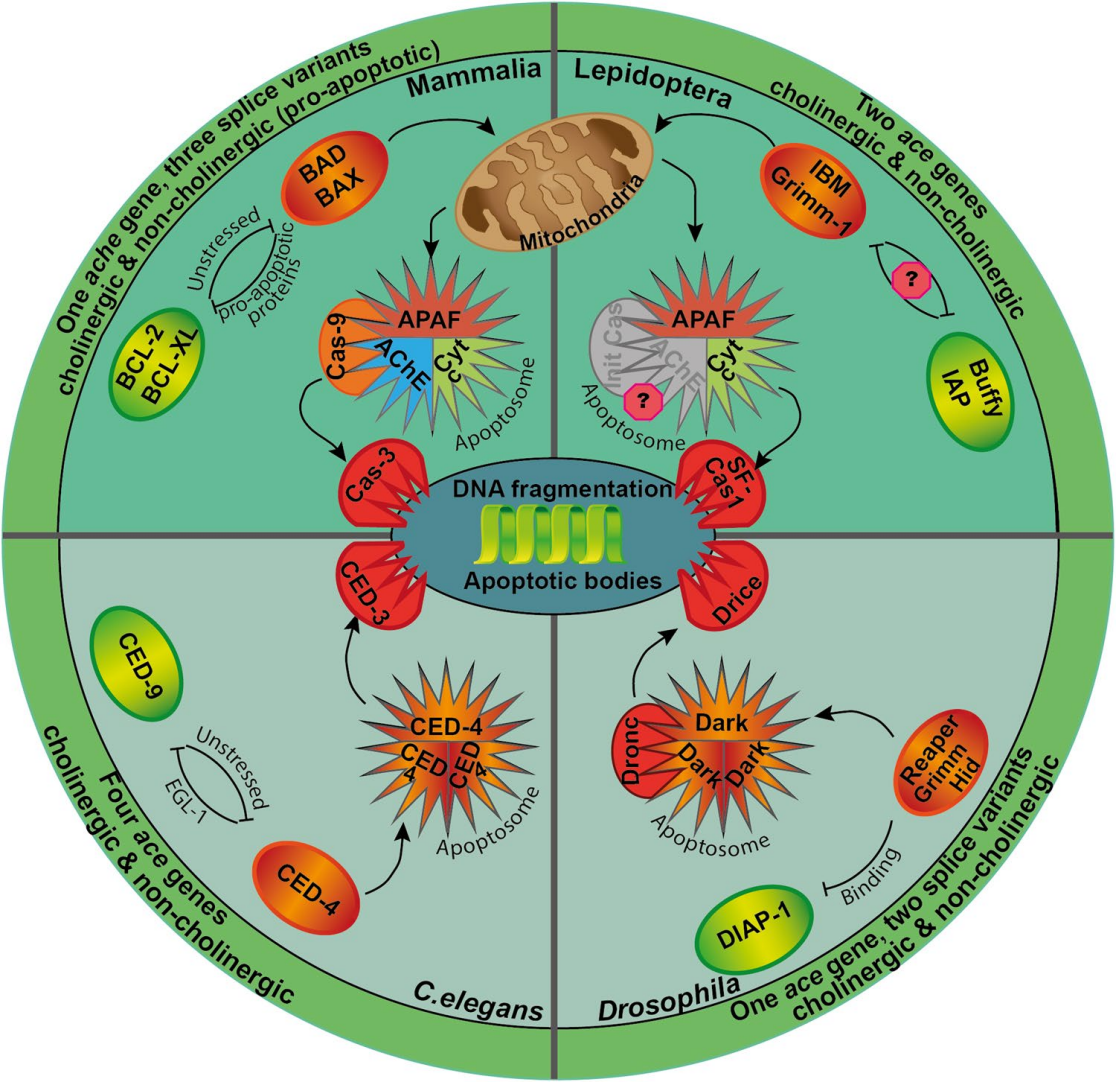
Apoptotic pathways in mammals, nematodes (*C. elegans*) and insects (*D. melanogaster* and lepidopteran species *S. frugiperda* and *B. mori*). Same colours depict similar functions mediated by orthologous or paralogous proteins. Top half of the circle depicts mitochondria-dependent apoptosis, bottom part illustrates mitochondria and cytochrome c independent apoptosis. Outer circle shows presence of *ache/ace* genes and their general functions in respective taxa. Mitochondria-dependent apoptosis is initiated by upregulation of pro-apoptotic proteins (BAD/BAX and IBM/Grimm-1; orange circles), release of cytochrome c into the cytosol, apoptosome formation (star-like structures) and activation of effector caspase (Cas-3 and SF-Cas 1) activation. AChE contributes to apoptosome formation in mammalia. Mitochondria- independent apoptosis is executed by upregulation/activation of pro-apoptotic agents (EGL-1/CED-4; Reaper/Grimm/Hid) that counteract anti-apoptotic factors (CED-9; DIAP-1 green circles). The apoptosome is formed either by accumulation of pro-apoptotic proteins (nematode; CED-4) or activation of an APAF-like protein (fly; Dark). The apoptosome will eventually lead to effector caspase activation (CED-3; Dronc). Both mitochondria dependent and independent pathways will result in DNA fragmentation, membrane blebbing and apoptotic body formation. *AChE* acetylcholinesterase, *APAF* apoptotic protease activating factor; *Cas* caspase; Cyt *c* cytochrome c, *IAP* inhibitor of apoptosis

Caspases that mediate the final stages of apoptosis cleave various selected substrates, either to destabilize or degrade structural elements of the cell or to activate executors of downstream apoptotic processes [9, 10] (Fig. 1). This may imply sequential activation of initiator caspases and executioner caspases (caspase-9 and caspase-3 in vertebrates; dronc and drice in *D. melanogaster*) or direct activation of executioner caspases (CED-3 in *C.elegans*). Caspases also perform various non-apoptotic functions [11] and are typically produced as enzymatically inactive zymogens [9]. Activation of caspases is mediated by non-caspase proteases (e.g. granzyme B in virally induced apoptosis) or weak intrinsic catalytic activity of procaspases (mammalian procaspase-8 with death-receptors; CED-4 oligomerization leading to CED-3 caspase activation in *C. elegans*). Formation of the apoptosome, a protein complex that associates in the cytosol as part of the intrinsic or mitochondrial apoptotic pathway, can also cause caspase activation (Reviewed in: [2, 12]). The vertebrate mitochondrial apoptotic pathway can be initiated by physiological stressors (hypoxia, toxins and others) that shift the balance of anti- and pro-apoptotic Bcl-2 proteins to the pro-apoptotic side by enhanced expression of BAX and BAD (Reviewed by [13]). This initiates the formation of mitochondrial outer membrane pores (MOMP) allowing cytochrome c (and other factors) release from the mitochondrial intermembrane space into the cytosol [14]. Cytochrome c associates with apoptotic protease activating factor-1 (Apaf-1) and procaspase 9. The resulting multi-protein complex, termed the apoptosome facilitates caspase 9 dimerization to release its function as initiator caspase that activates effector caspase-3 [15, 16]. While apoptosome formation in *C. elegans* and most cell types of *D. melanogaster* is independent from mitochondria-derived factors [17] (Fig. 1), contribution of cytochrome c has been described in lepidopteran species [18, 19] and two cell types (interommatidial retina cells and sperm cells) of *D. melanogaster* [20, 21].

Promotion of apoptosis by acetylcholinesterase (AChE) has been demonstrated in several studies on various mammalian cell types, suggesting that AChE is commonly involved in apoptosis regulation (Review: [22]). Generally, AChE is known to terminate synaptic transmission at cholinergic synapses by hydrolytic cleavage of the transmitter acetylcholine. However, other non-canonical functions of AChE in cell growth, cellular differentiation, cell adhesion and amyloid fiber assembly (amongst others) have been reported [22–24]. Vertebrates express a single *AChE* gene by different cell types in various tissues either constitutively or stimulus-induced. Alternative splicing leads to three major variants with identical esterase domains and distinct carboxy-terminal domains that seem to determine specific biological and pathological functions [22, 25–27]. Expression of the synaptic form of AChE is increased in response to apoptotic stimuli [27–29]. The presence of AChE per se is not sufficient to induce apoptosis. However, overexpression of AChE has been demonstrated to sensitize cells towards apoptosis induction [30] while suppressing AChE expression or pharmacological inhibition of its activity can rescue cells from apoptotic death [23, 28, 29, 31–33]. Both cytosolic appearance and translocation into the nucleus have been associated with the execution of apoptosis (see below). Nevertheless, all isoforms of AChE are translated in the endoplasmic reticulum and are designated to become intramembraneous, membrane attached or secreted proteins. To our knowledge, the mechanisms by which AChE can accumulate in the cytosol remain unresolved (see [34] for a general discussion). In the cytosol, AChE interacts with components of the apoptosome including Apaf-1 and cytochrome c [35] and suppression of AChE expression inhibits apoptosome formation [31]. A detailed study on rat PC12 cells [36] demonstrated an increased AChE expression following apoptosis induction. Additionally, a caspase-mediated cleavage of cytosol-located AChE at its N-terminus and a subsequent translocation of cleaved and full-length AChE into the nucleus were observed. Prevention of AChE cleavage by caspase inhibitors or stimulation of Akt signalling interfered with apoptotic cell death [36]. Accumulation of AChE in the nucleus of apoptotic cells has been described in several studies on different cell types [28, 30, 37]. AChE has been shown to cleave DNA in nuclei of apoptotic cells independent of other nucleases [32]. Hence, AChE seems to promote apoptosis in mammalian cells by contributing to cytochrome c-triggered apoptosome formation and degradation of nuclear DNA.

Involvement of AChE in regulation of apoptosis has not been described for invertebrate species. Insects, with the exception of cyclorrhaphan flies including *Drosophila melanogaster,* express two AChE genes, *ace-1* and *ace-2* [38–41]. Depending on the species, either *ace-1* or *ace-2* is expressed at higher levels and serves as the major enzyme that hydrolyzes synaptically released acetylcholine. The other AChE has frequently been described to perform non-synaptic functions involved in the regulation of growth, reproduction and development [often described as “non-cholinergic functions” [38, 41–43]]. Since acetylcholine is a major transmitter of sensory neurons and excitatory central nervous system synapses in insects, a large number of pharmacological agents used for pest control target AChE activity [43, 44]. Though AChE expression in non-neuronal cells has been reported [45], non-synaptic functions of insect AChE have not been resolved in detail.

In this study we challenged primary brain cell cultures from the migratory locust *Locusta migratoria* by exposure to hypoxic conditions. Hypoxia-induced cell death was accompanied with the characteristic phenotypic alterations attributed to apoptosis. We demonstrate increased *ace-1* expression in vivo during hypoxia exposure and significant reduction of hypoxia-induced cell death in the presence of two different AChE inhibitors in vitro. These results indicate that AChE promotes apoptosis in locusts, as it has previously been described for mammalian cells. This similarity provides another piece of evidence for the presence of a complex “mammalian-like” apoptosis regulatory system in the evolutionary ancestors of mammals and insects.

## Methods

Studies were performed with fifth instar nymphs of *Locusta migratoria.* Animals were purchased from a commercial breeder (HW-Terra; Herzogenaurach, Germany) and maintained at 24 °C; 55% humidity with a 12/12 h day/night cycle**.**

### Locust primary neuron culture

Primary neuron cultures were prepared as described previously [46–48]. In brief, two juvenile locust brains were dissected per primary culture. All brains of the same experiment were pooled and washed three times in Leibowitz 15 medium (L15; Gibco, Life Technologies, Darmstadt, Germany) supplemented with 1% Penicillin/Streptomycin (P/S; 10,000 units/ml penicillin and 10 mg/ml streptomycin, Sigma-Aldrich, Munich, Germany) and Amphotericin B (Ampho; Gibco, 250 µg/ml, ThermoFisher Scientific, Germany). Pooled brains were treated with Collagenase/Dispase (2 mg/ml; Sigma-Aldrich, Munich, Germany) for 30 min at 27 °C. Enzymatic digestion was stopped by three washes in Hanks balanced salt solution (Gibco, Life Technologies, Darmstadt, Germany). Brain cells were mechanically dissociated in 1 ml L15 by repeated pipetting. The dissociated neurons were briefly spun down with a table top centrifuge and the supernatant was discarded. The cell pellet was resuspended in 100 µl medium per cell culture. The cell suspension was equally distributed onto Concanavalin A-coated coverslips (ConA; Sigma-Aldrich, Munich, Germany). The coverslips (Ø 1 cm; Hartenstein, Würzburg, Germany) were coated for 1 h at room temperature, subsequently washed three times in PBS and placed into the centre of a culture dish (Ø 3 cm; Corning, New York, USA). Cells were let to settle down and attach to the coverslip for 2 h at room temperature before filling the dish with 1.9 ml L15 medium. Culture medium was further supplemented with 5% fetal bovine serum gold (FBSG; PAA Laboratories GmbH, Pasching, Austria) for the first four days. Cultures were maintained in humidified normal atmosphere at 27 °C if not stated otherwise. Culture medium was exchanged every other day.

### AChE inhibition and hypoxia treatment

To evaluate the effect of AChE inhibition on cell survival in normal culture conditions, cell culture medium was supplemented with either 10 µM neostigmine bromide (NSB; dissolved in H_2_O; Sigma-Aldrich, Munich, Germany) or 10 µM and 1 µM territrem B (TRB, initially dissolved in methanol and further diluted in cell culture medium [MeOH final concentration < 0,001%]; Abcam, Cambridge, United Kingdom) for four days beginning at culture establishment. In order to evaluate the impact of AChE-inhibition on stressed neurons, cell cultures were exposed to hypoxic conditions for 36 h (0.3% O_2_; Hypoxia Incubator Chamber, STEMCELL™, Cologne, Germany). Cultures with and without supplemented AChE inhibitor were initially maintained for 5 days. On in vitro day 5, two cultures (AChE inhibitor-treated and untreated) were exposed to hypoxia, while one equivalent culture remained in normoxic conditions at the same temperature as the hypoxia-exposed cultures. Hypoxia-exposed cultures were reoxygenated for 12 h and all cultures were subsequently fixed in 4% paraformaldehyde (PFA).

### Cell viability assessment

Cell survival was assessed by DAPI nuclear staining. This method to distinguish physiologically intact from dead or dying neurons was validated by comparison with testing membrane integrity with the trypan blue exclusion assay [49] and TUNEL staining [this study]. Fixed cells were washed three times in PBS before washing twice with PBS/0.1% Triton-X-100 (PBST). Cultures were incubated for 30 min in DAPI (Sigma-Aldrich; Munich, Germany; 1:1000 in PBST) in the dark. Subsequently, coverslips were washed five times in PBS and once in DABCO (Roth, Kalsruhe, Germany) before mounting in DABCO. Experimental groups were evaluated with an epifluorescence microscope (Zeiss Axioskop; 40 × objective, Oberkochen, Germany) equipped with a Spot CCD camera (Invisitron, Puchheim, Germany). Two rows of non-overlapping photographs (~ 80 on average) covering the entire extension of the coverslips were taken from each cell culture for subsequent analysis. Live/Dead assessment was performed by manual counting of DAPI stained nuclei by an observer who was blinded with respect to the culture treatment. Cell counting was supported by using ImageJ Cell counter plug-in (Fiji ImageJ by NIH) as described elsewhere [46–48].

### Statistical analysis

Data of individual experiments were normalized to untreated control cultures with neurons derived from the same pool of locust brains, to evaluate the relative portion of surviving cells per culture. Data was analysed using RStudio (Version 1.2.1335 [50, 51]) employing pairwise permutation test (included in packages “coin” and “rcompanion” [52–54]). Data are represented in box plots including the median, upper and lower quartile. Whiskers represent 1.5 × interquartile range. Circles show single data points. Benjamini–Hochberg correction was performed to avoid false positives resulting from multiple comparisons.

### Anti-cleaved caspase-3 immunostaining

Locust primary neuron cultures were stained for the presence of cleaved caspase-3, the activated form of caspase-3. Cultures were established and stressed by hypoxia as described above. For comparison of morphological alterations, apoptosis was additionally induced by exposing cultured locust neurons to UV light (10 h; TL-D 25 W G13, Philips Health Systems, Hamburg, Germany) or Mitomycin C (60 µg/ml, Sigma-Aldrich, Munich, Germany). After fixation in 4% PFA, cells were washed in PBS and subsequently permeabilized in PBST (0.1%). Coverslips where blocked in blocking solution (PBS/0.1% Triton; 5% normal goat serum; 0.25% bovine serum albumin) for 1 h before incubation with α-rabbit cleaved caspase-3 antibody (1:300 in blocking solution; Calbiochem, Merck, Darmstadt, Germany) at 4 °C over night. Subsequently, coverslips were washed in PBS before applying the secondary antibody (Cy2 goat- α-rabbit; 1:200 in blocking solution; Dianova, Hamburg, Germany) and DAPI (1:1000) for 2 h at room temperature. Cells were washed with PBS before mounting with PBS/Glycerin (1:1). Images of cleaved caspase-3 associated immunofluorescence were taken with Leica TCS SP8 (40 × magnification; Leica Microsystems, Wetzlar, Germany).

### Terminal deoxynucleotidyl transferase dUTP nick end labelling (TUNEL) staining

Locust primary neuronal cell cultures were prepared and stressed as described above. TUNEL stainings were performed using the Abcam In situ Direct DNA Fragmentation (TUNEL) Assay Kit (Abcam, Cambridge, United Kingdom) according to manufacturer’s instructions. However, all staining steps were performed on cells attached to coverslips and volumes were adjusted to well sizes. Analysis of propidium iodide nuclear staining (488/623 wavelength) and fluorescein-labelled DNA fragments (488/520 wavelength) was performed with a Leica SP8 confocal microscope (Leica Microsystems, Wetzlar, Germany).

### Acetylcholinesterase-activity stainings in locust brain slices

NSB- and TRB-mediated interference with AChE activity was demonstrated on fixed brain sections of *L. migratoria* as originally described by Karnovsky and Roots [55] and modified for insect brain sections by Hoffmann and colleagues [56]. Briefly, dissected brains were fixed for 2 h in a mixture of 2.5% glutharaldehyde and 4% paraformaldehyde diluted in phosphate buffer, embedded in 5% agarose and sectioned horizontally (with respect to neuraxis) with a vibrating blade microtome (40 µm; VT 1000 S, Leica, Wetzlar, Germany). Brain slices were permeabilized with detergent, washed in Tris-Maleic buffer (TMB, pH 6) and one portion was incubated in NSB or TRB (10 µM, 1 µM and 0,1 µM respectively) diluted in TMB for 30 min. Control sections were incubated in TMB during that time. Subsequently, all but some sections, separated as negative control (no staining expected), were incubated for 45 min in freshly prepared AChE activity staining solution containing 10 mg acetylthiocholine iodide, 29.4 mg 0.1 M sodium citrate, 7.5 mg 30 mM CuSO_4_, and 1.6 mg 5 mM K_3_(Fe(CN)_6_) dissolved in 7.5 ml Tris-maleate buffer, with or without AChE inhibitor. After repeated washing in TMB brain sections were mounted on microscopy slides in DABCO and analysed by light microscopy.

### DNA isolation and DNA ladder

Locust primary neuronal cell cultures were prepared and stressed as described above. DNA was isolated following the protocol of Kasibathla and colleagues [57]. 1.5% Agarose gels were run at 50 V for 2 h. DNA was visualized by Roti®-GelStain (Roth, Karlsruhe, Germany) and documented with an iBright CL1500 Imaging System (Thermo Fisher Scientific, Osterode am Harz, Germany).

### qRT-PCR analysis of in vivo *ace-1* expression

Intact juvenile locusts were exposed to hypoxic conditions (0.3% O_2_) for either 6.5 or 24 h while control animals were maintained in normoxic conditions for identical periods. For transcript expression analysis, brains were dissected either immediately after the end of the hypoxic period or after 1 h reoxygenation in normal atmosphere. Typically, five brains per treatment were pooled and RNA was extracted using Trizol (Sigma-Aldrich, Munich, Germany). Brain tissue was lysed in Trizol by TissueLyser LT (Qiagen, Hilden, Germany) aided by a 3 mm stainless steel bead**.** 200 µl chloroform (Labsolute, Th. Greyer, Renningen, Germany) were added and the samples were vigorously shaken for 15 s. Subsequently, lysed tissue samples were placed on ice for 15 min before centrifuging for 15 min at 12.000 × g at 4 °C. The RNA-containing translucent phase was carefully transferred to a fresh tube and precipitated with ice-cold 75% ethanol. Samples were incubated for at least 30 min at − 20 °C before centrifuging for 10 min at 10.000 × g at 4 °C. The resulting RNA-containing pellet was washed three times in cold 75% ethanol before drying and elution in 30 µl ddH_2_O. RNA concentration was measured with NanoDrop™ (Thermo Scientific, Schwerte, Germany)**.** Prior to cDNA synthesis RNA was subjected to DNase treatment using DNA-*free*™ DNA Removal Kit (Invitrogen, Schwerte, Germany; #AM1906) according to manufacturer’s instructions. The same protocol was used to extract RNA from control animals that were continuously maintained in normoxic conditions.

cDNA was synthetized using LunaScript™ RT SuperMix Kit (New England BioLabs, Ipswich, MA, USA) according to the manufacturer’s instructions. All reverse transcriptions were performed with 1 µg RNA as template.

qRT-PCR primers specific for locust *18 s* *rRNA* and *gapdh* were designed according to the corresponding sequences (*18 s* *rRNA* AF370793; *gapdh* JF915526). Lm-*ace* sequence of 560 bp was identified by aligning *Locusta migratoria manilensis* sequence (EU231603) and *Tribolium castaneum* (*ace-1* HQ260968; *ace-2* HQ260969) sequences against locust genome available on i5k platform (https://i5k.nal.usda.gov/locusta-migratoria). Alignments were performed using blastn with default settings, implemented on i5k platform*.* Sequence similarities were computed using *Geneious Prime®* (Version 2019.2.3) and ClustalW alignment tool (default settings applied). Computed *Lm-ace-1* sequence, alignments of reference sequences and sequence similarities are shown in Appendix Fig. 6, Table 3 and 4.

Prior to experimental data collection, all primers were tested for efficiency and housekeeping genes (HKG) were further tested for stable expression in hypoxic samples (See Appendix Fig. 7 and Table 5). Primers used for qRT-PCR are summarized in Table 1.

**Table 1:** qRT-PCR oligonucleotides used in this study

Gene	Sequence from 5′-3'	Accession no
*Lm-18S fwd*	CATGTCTCAGTACAAGCCGC	AF370793
*Lm-18S rev*	TCGGGACTCTGTTTGCATGT	
*Lm-gapdh fwd*	GTCTGATGACAACAGTGCAT	JF915526
*Lm-gapdh rev*	GTCCATCACGCCACAACTTTC	
*Lm-ace fwd*	TTTGAAATGGCGGTGGTAGC	Computed sequence
*Lm-ace rev*	GTCGGAGGACTGCCTGTAC	

*Gapdh* primers were previously published in [48]

qRT-PCRs were run using the MyiQ™ Single-ColorReal-Time PCRDetection System (Bio-Rad, Munich, Germany) in a 96-well plate with final PCR reaction volumes of 5 µl Luna® Universal qRT-PCR Master Mix (New England BioLabs, Ipswitch, MA, USA), 0.1 µM forward and reverse primer and 10 ng cDNA (10 µl final reaction volume). Samples were run as triplicates and ( −) RT controls were added for all measurements. Table 2 shows the program for cDNA amplification. Data was analysed using the Pfaffl method [58] and the geometric means of both *Lm-18S* and *Lm-gapdh* normalized values was calculated and plotted as bar plots in RStudio.

**Table 2:** qRT-PCR program for amplification of *ace-1*, *18 s* rRNA and *gapdh* in locust brain cDNA samples

Step	Time [s]	Temperature [°C]	
Initial denaturation	180	95	
PCR reaction			
Denaturation	10	95	40×
Annealing	30	60	
Elongation	30	72	
Melting curve			
Denaturation	60	95	
Annealing	60	55	
Melting curve	10	55	0.5 °C per cycle up to 95 °C

## Results

### Characterization of locust neuronal apoptosis

**Fig. 2:**
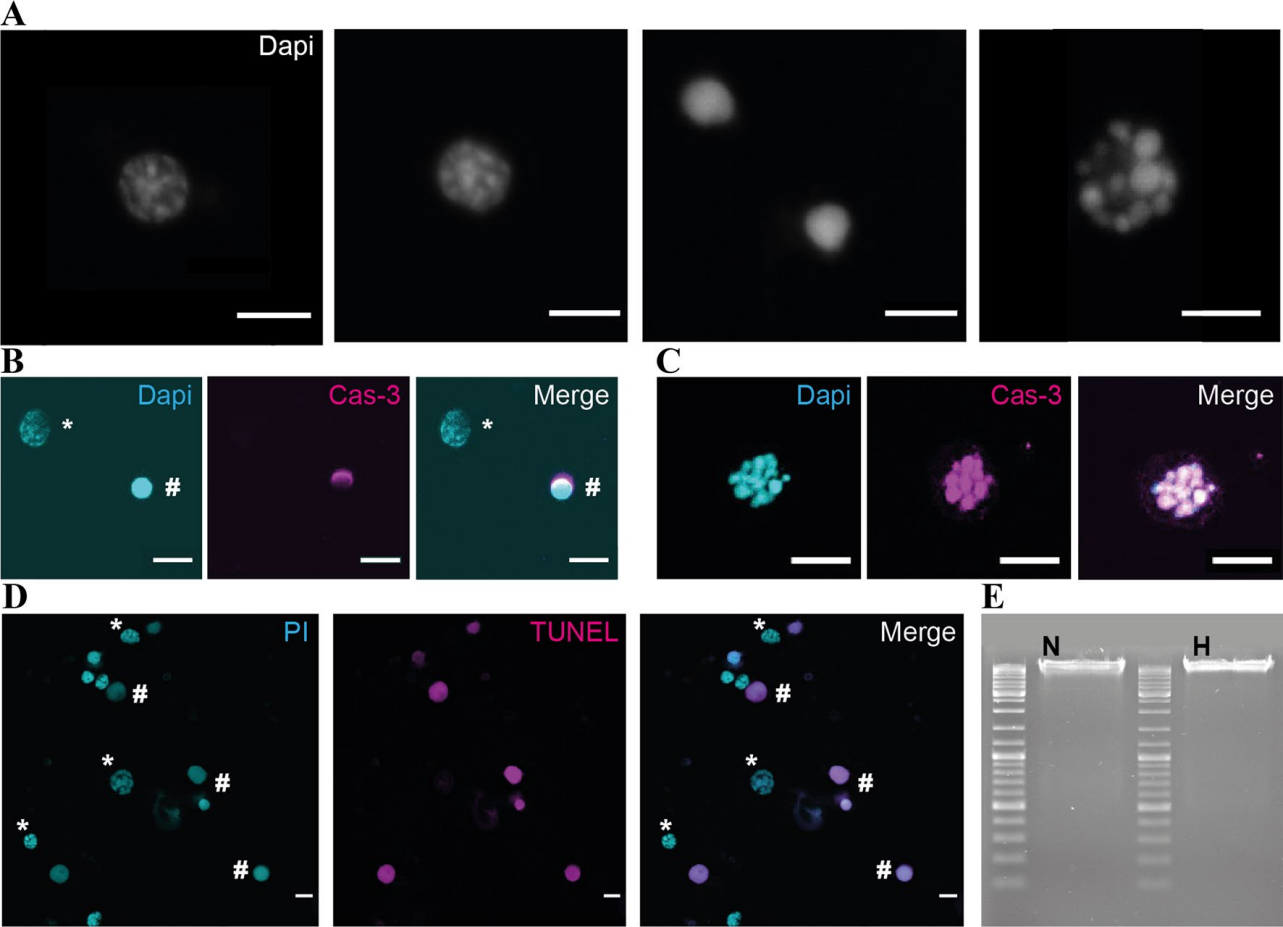
Characterization of locust neuronal apoptosis. **a** DAPI nuclear staining illustrates the process of DNA condensation in dying locust neurons. From left to right: Nucleus of intact cell. Condensation of chromatin structure, distinguishable by more tightly packed nuclear organization. Fully condensed DNA in two nuclei. Nucleolysis leads to formation of apoptotic bodies. Scale bars 5 µm. **b** Nuclei of one intact (*) and one dead (#) neuron with DAPI labelling, α-cleaved caspase-3 immunofluorescence and merged signals. Cleaved caspase-3 activity is visible as a halo on the nuclear envelope of the dead cell. Scale bars 10 µm. **c** DAPI labelling of a nucleus with beginning nucleolysis. α-cleaved caspase-3 immunofluorescence and merged signals that almost completely coincide. Scale bars 10 µm. **d** DNA fragmentation of neurons visualized by TUNEL assay. Nuclei of intact (*) and dead (#) neurons labelled with propidium iodide, TUNEL staining of DNA double strand breaks and merged signals. Only cells with condensed chromatin contain TUNEL staining. Scale bars 10 µm. **e** After separation of DNA from hypoxia-exposed locust neurons on 1.5% agarose gel no DNA fragmentation (“DNA ladder”) is detectable. N = Normoxic sample, H = 36 h hypoxia sample. 1 kb DNA ladder used as reference

In order to characterize hypoxia-induced death of locust neurons as apoptotic, primary neuron cultures were subjected to cleaved-caspase-3 immunostaining, DNA labelling with DAPI and TUNEL stainings. First, we aimed to identify different stages of chromatin condensation in stressed neurons. Figure 2a displays a timeline (from left to right) of neuronal DNA condensation during apoptosis. Healthy cells display characteristic DAPI staining of intact euchromatin structures. During apoptosis, volumes of nuclei shrink and chromatin gets increasingly condensed until uniform DAPI labelling extends over the entire nucleus. In most cases, condensed nuclei persisted after cytomembranes disintegrated and the cytosol with all organelles was released into the culture medium. Only exceptionally nucleolysis and the generation of apoptotic bodies were observed (Fig. 2a, right). Additionally, we performed immunofluorescent stainings of cleaved-caspase-3 in hypoxia exposed neurons. Figure 2b shows representative stainings of an intact and a dead cell. The intact cell, recognizable by the typical patchy DAPI labelling pattern of its nucleus, lacks any cleaved-caspase-3 related immunoreactivity. Caspase-3 activity can be observed along parts of the nuclear envelope of the dead cell. Furthermore, nuclei which were in the process of apoptotic body formation displayed immunoreactivity in all nuclear fragments (Fig. 2c).

TUNEL stainings were conducted in order to visualize DNA double-strand breaks that typically appear during apoptotic DNA degradation. Figure 2d shows TUNEL positive staining in dead cells with highly compacted DAPI-labelled chromatin structure, while all intact cells with patchy DAPI-related fluorescence lacked the TUNEL related signal. Generally, neuron cultures not exposed to hypoxia displayed fewer cells with TUNEL positive nuclear staining in comparison with stressed cells (data not shown). DNA ladder formation as a sign for extensive internucleosomal DNA cleavage was not detected, when isolated DNA from stressed in vitro samples was separated by electrophoresis (Fig. 2e).

Besides hypoxia, primary cultured locust neurons were exposed to two additional stressors, that induced apoptosis in other studies. Locust neurons were either exposed to 10 h of UV light or to 60 µg/ml Mitomycin C (MMC) for 24 h. UV light was shown to induce DNA breaks followed by apoptotic death in a cell line derived from lepidopteran ovaries [59]. MMC is generally used in mouse embryonic fibroblast cultivation in order to disrupt DNA replication. Studies have shown that high concentrations of MMC can damage DNA and cause apoptosis in post-mitotic cells [60].

Both stressors induced equal cellular changes as hypoxia, including caspase-3 activation, nuclear condensation and DNA fragmentation in dying and dead cells’ nuclei visualized in TUNEL stainings (summarized in Suppl. Fig. S8). Also similar to hypoxia-induced apoptosis, DNA fragmentation leading to DNA ladder formation was not detectable.

### Increased expression of *ace-1* in hypoxia-exposed locust brains

A sequence with high (99.5%) similarity to *L. migratoria manilensis ace-1* was detected by blastn search in the poorly annotated genome of *L. migratoria migratoria* (Suppl. Fig. S6). Comparison of the identified *L. m. migratoria* consensus sequence with reported sequences from *T. castaneum* revealed a higher similarity with *Tc-ace-1* (60.3% coverage) than with *Tc-ace-2* (46.7% coverage). Blast search using *Tc-ace-1* revealed high sequence similarity in the *L. m. migratoria* genome (73% coverage) in the same region that corresponded to the *L. m. manilensis* sequence. *Tc-ace-2* blast identified a corresponding sequence in the *L. m. migratoria* genome in a different region (76% coverage). These results suggest that the *L. migratoria ace* sequence we have been targeting is *ace-1*. Sequence similarities are summarized in Tables 3 and 4.

**Fig. 3:**
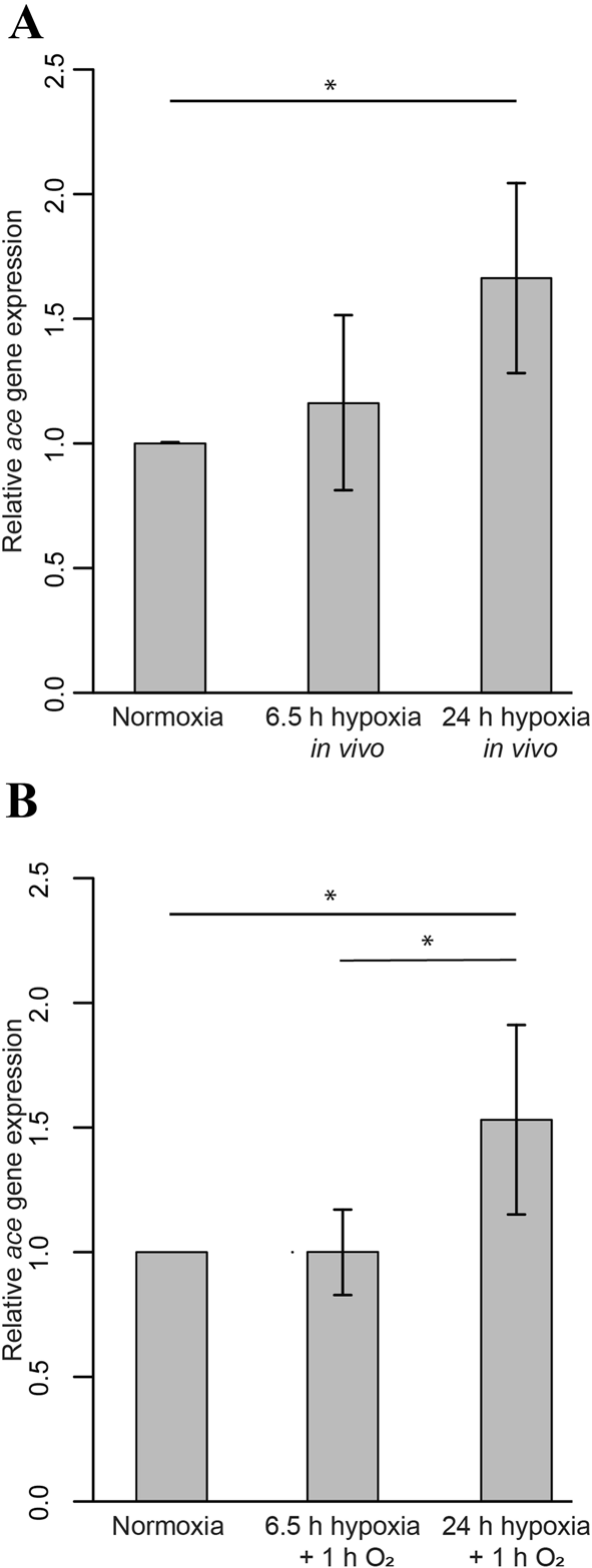
In vivo expression of *Lm-ace-1* transcript in hypoxia-exposed juvenile locusts. **a** Juvenile locusts were exposed to either 6.5 or 24 h hypoxia followed by immediate RNA isolation from dissected brains. qRT-PCR analysis shows minor insignificant changes after 6.5 h (1.19 ± 0.36 STDV) and significant (p = 0.02) moderate upregulation after 24 h (1.66 ± 0.38 STDV). **b** Juvenile locusts were exposed to either 6.5 or 24 h hypoxia followed by 1 h reoxygenation in normal atmosphere before RNA isolation from dissected brains. Average levels of *ace-1* transcript are unchanged (1.001 ± 0.1 STDV) after 6.5 h plus reoxygenation and moderately elevated (1.54 ± 0.39 STDV) after 24 h hypoxia plus reoxygenation in comparison with normoxic controls and 6.5 h exposed animals (both p = 0.01). **a**, **b** *18 s* *rRNA* and *gapdh* were used as internal controls. Geometric mean from n = 6 (except n = 3 for 6.5 h hypoxia plus reoxygenation in 3B) experiments was calculated and data plotted with RStudio. Statistics calculated with pairwise permutation test and Benjamini–Hochberg correction

Juvenile locusts were exposed to either 6.5 or 24 h hypoxia (0.3% O_2_) before RNA isolation from dissected brains. qRT-PCR analysis, using both *Lm-18S rRNA* and *Lm-gapdh* as reference genes revealed a minor increase of average *ache* expression in animals exposed for 6.5 h to 1.19 (± 0.36 STDV) fold of expression in untreated controls (Fig. 3a). Average *ace-1* expression levels increased to 1.66 (± 0.38 STDV) fold of control levels after 24 h exposure to hypoxia (significantly different with p = 0.02). To identify if *ace-1* transcription was hampered due to insufficient ATP as a result of hypoxic conditions, animals were exposed to both 6.5 and 24 h hypoxia followed by a 1 h reoxygenation period before RNA extraction. During the reoxygenation period locusts exposed to 6.5 h hypoxia reassumed upright position and moved spontaneously. Animals exposed to 24 h hypoxia only performed twitches with their legs but did not reassume upright position. As shown in Fig. 3b, average *ace-1* expression levels remained unchanged after 6.5 h hypoxia plus reoxygenation (1.001 ± 0.1 STDV) and were slightly elevated after 24 h hypoxia plus reoxygenation (1.53 ± 0.39 STDV) compared to untreated control animals and 6.5 h exposed animals (both significantly different with p = 0.01). Therefore, the average of *ace-1* transcript was slightly reduced during the 1 h reoxygenation period (compare Figs. 3a and 3b).

### AChE inhibition increases the survival of insect neurons in vitro

**Fig. 4:**
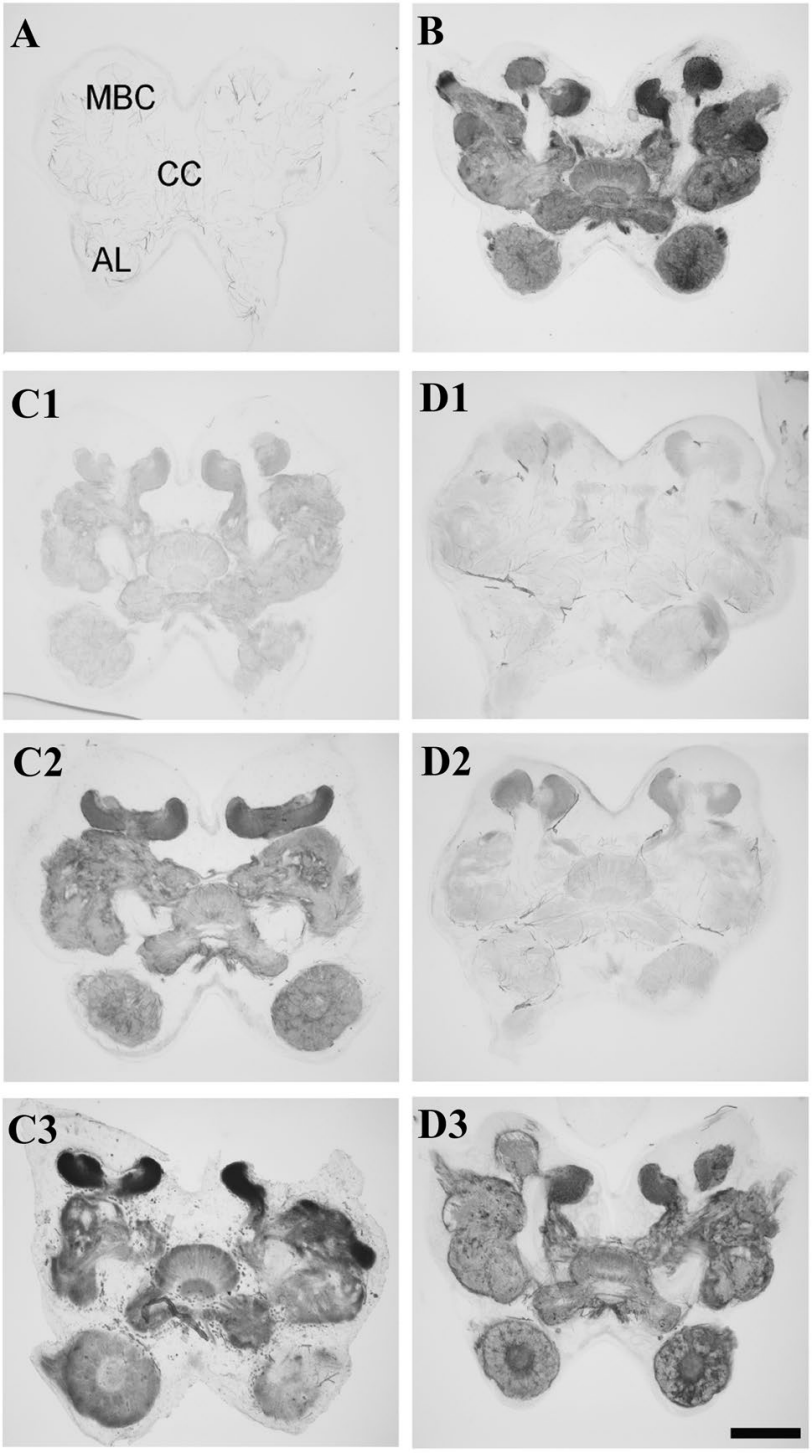
Acetylcholinesterase activity staining in locust brain slices. **a** Negative control incubated without substrate solution. Letters indicate position of neuropils that appear stained in B, C and D (AL antennal lobes, CC central complex, MBC mushroom body calyces). **b** Positive control incubated in substrate solution without AChE inhibitor. Strong AChE reaction product accumulates in various brain neuropils. C, D: Brain slices incubated for 45 min in substrate with different concentrations of NSB (C) and TRB (D). C1, D1: 10 µM. C2, D2: 1 µM. C3, D3: 0.1 µM. Stainings indicate a concentration-dependent inhibition of AChE activity-generated staining by both AChE inhibitors. Scale bar 250 µm valid for all photographs

As a first step, we confirmed that neostigmine bromide (NSB) and territrem B (TRB) inhibit the hydrolysing activity of locust AChE. Fixed brain sections were incubated with acetylthiocholine (as part of a staining solution developed by Karnovsky and Roots [55]), which is converted into a dark precipitate by AChE. As shown in Fig. 4b, AChE-associated precipitate accumulated in various brain neuropils known to receive cholinergic innervation, including antennal lobes, central complex and mushroom body calyces. In contrast, brain sections that were not exposed to the staining solution remained entirely unlabelled (Fig. 4a). Similarly, AChE-generated precipitate was largely absent in brain sections that were co-exposed to staining solution and 10 µM NSB (Fig. 4c1) or 10 µM and 1 µM TRB (Fig. 4d1, 2). Little precipitate was detected in antennal lobes, central complex and most other protocerebral neuropils, while slightly enhanced staining developed in the mushroom body calyces, which are known to receive profound cholinergic innervation. Nevertheless, largely reduced staining indicated that 10 µM NSB and 1 µM and 10 µM TRB successfully suppressed most of the AChE-mediated conversion of the substrate. Stronger staining developed in the presence of 1 µM and 0.1 µM NSB (Fig. 4c2, 3) and 0.1 µM TRB (Fig. 4d3) indicating weak or absent AChE inhibition at these concentrations. At 10 µM and 1 µM concentration TRB inhibited AChE activity more potently than NSB.

**Fig. 5:**
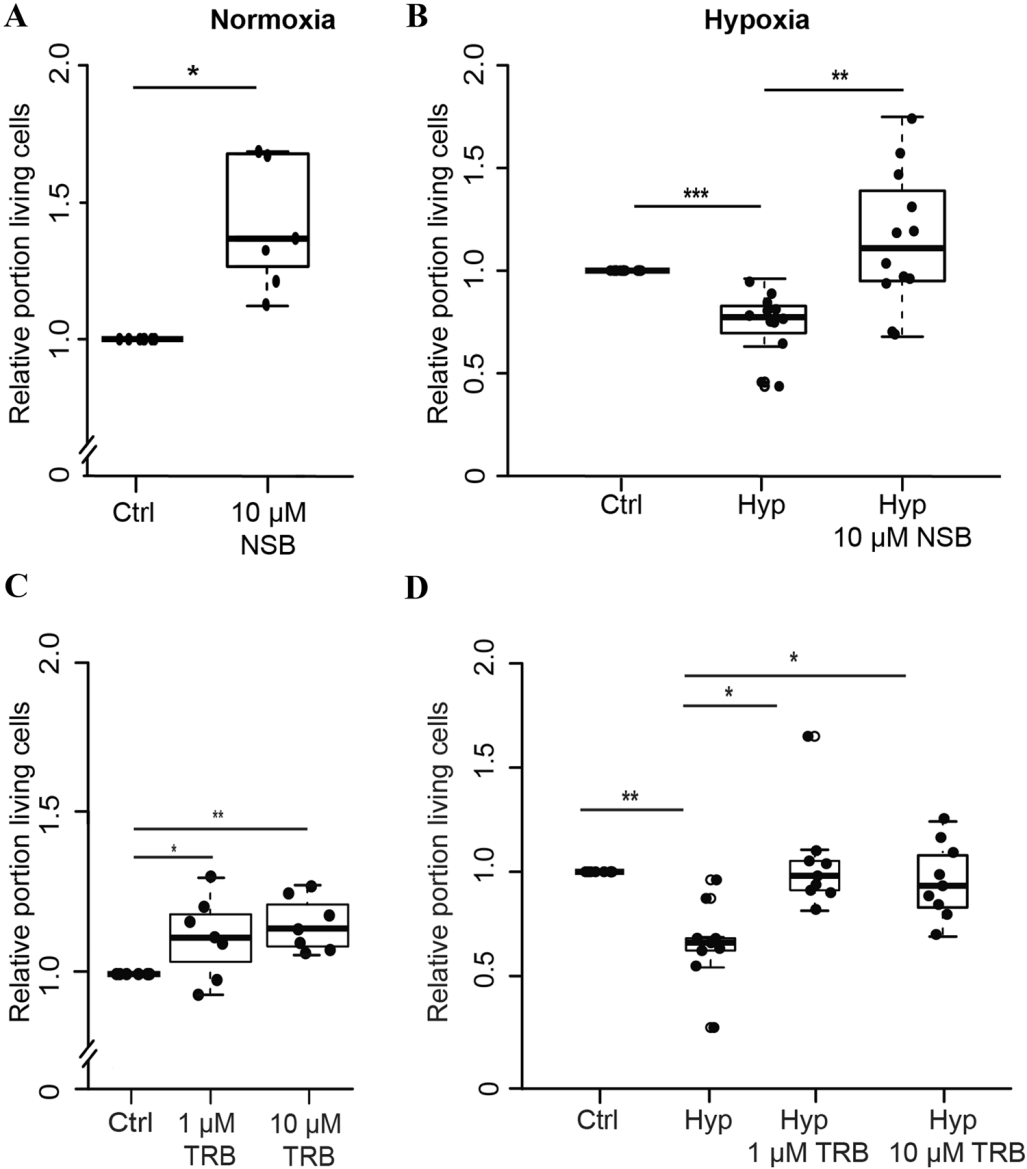
AChE inhibition increases neuronal survival in vitro. **a** Relative survival of locust neurons after 4 days in vitro. Inhibition of AChE with 10 µM NSB significantly increased neuronal survival in normoxic control conditions. n = 7; 31.029 cells evaluated. **b** Relative survival of locust neurons after exposure to hypoxia (O_2_ < 0.3%) for 36 h. Hypoxia-exposure reduced the median relative survival to 70% compared to control cultures that derived from the same pool of locust brains. Presence of 10 µM NSB increased the survival of hypoxia-exposed neurons in each experiment. In some experiments, relative survival was even higher than in normoxic control cultures resulting in a median relative survival of 1.2 (compared to the controls in normoxic culture conditions). Cells were maintained in culture for five days before exposure to hypoxia. n = 12; 66.802 cells evaluated. **c** Effect of 1 µM and 10 µM TRB on locust neuronal survival in normoxic conditions. Treatment with either TRB concentration significantly increased cell survival in comparison to control. n = 7; 56.099 cells evaluated. **d** Relative survival of locust neurons exposed to 36 h hypoxia (O_2_ < 0,3%). Hypoxia reduced the median cell survival to 60% in comparison to the control. Pretreatment with both 1 µM and 10 µM TRB rescued cells from hypoxia-induced apoptosis. n = 9; 95.907 cells evaluated. All statistics calculated with pairwise permutation test and Benjamini–Hochberg correction. * p < 0.5, ** p < 0.01, *** p < 0.001

Initial experiments assessed the principal survival of primary neuron cultures from locust brains in the presence of the AChE inhibitor NSB. Primary neurons were cultured in full medium (with serum) supplemented with 10 µM NSB (concentration was chosen according to histological experiments described above), starting immediately after culture establishment. After four days in vitro, cell survival was quantified on the basis of DAPI nuclear morphology and normalized to the respective untreated control cultures, which derived from the same pool of locust brains as the NSB-treated cultures. Comparison of non-treated control cultures with NSB-treated cultures revealed increased survival in the presence of the AChE inhibitor in all seven experiments (Fig. 5a; 12 – 63% increase of surviving neurons; significantly different with p = 0,02). These results indicate that NSB supports the survival of locust primary neurons under normal culture conditions.

In order to evaluate whether inhibition of AChE alters neuronal survival under challenging physiological conditions, locust primary neuron cultures were subjected to hypoxia (O_2_ < 0,3%) for 36 h. Data was normalized to the respective control culture maintained in normoxic conditions, that derived from the same pool of locust brains as the respective experimental cultures. Hypoxia exposure reduced neuronal survival to 40 – 90% of survival in normoxic controls in all 12 experiments leading to a highly significant (p = 0.0005) loss of intact cells (Fig. 5b). Locust neurons that were maintained in medium supplemented with 10 µM NSB were significantly less sensitive to hypoxia-induced apoptosis (p = 0.003). Within each experiment, relative survival in hypoxic NSB-treated cultures was higher than in the hypoxia-exposed cultures, derived from the same locust brains. In seven out of 12 experiments, survival in NSB-treated hypoxic cultures was even higher than in respective normoxic control cultures, although overall neuronal survival was not significantly different between these groups. These results indicate that the AChE inhibitor NSB interferes with hypoxia-induced apoptosis of locust primary brain neurons.

In order to confirm that the observed antiapoptotic effects of NSB resulted from its interaction with AChE, we exposed cultured locust neurons to another AChE inhibitor, TRB, with a different molecular structure and mode of AChE inhibition. Since 10 µM and 1 µM TRB inhibited AChE activity in locust brain sections, we used both concentrations in these experiments. TRB increased the survival of locust neurons under normal culture conditions (Fig. 5c). Compared to control cultures in normal medium, 1 µM TRB increased cellular survival (p = 0.03) in five out of seven experiments (range of relative survival in all experiments: 98 – 130%, average: 111%). TRB in the concentration of 10 µM increased cellular survival in all seven experiments (106 – 127%; average: 115%, p = 0.004). TRB also protected locust neurons from hypoxia-induced cell death (Fig. 5d). Compared to normoxic control cultures, hypoxia significantly reduced the average cell survival to 68% (p = 0,001). Treatment with 1 µM and 10 µM TRB increased the mean cell survival in hypoxia close to the level of survival in normoxic control cultures (105% and 99% respectively relative survival). Compared to hypoxia-exposed cultures, significantly more neurons survived in the presence of both concentrations of TRB (1 µM: p = 0.004, 10 µM: p = 0.007), indicating that TRB interferes with hypoxia-induced death of primary locust brain neurons.

## Discussion

Apoptosis is involved in development, disease- and pathogen-induced cell death and tissue responses to challenging situations in most if not all animals. Prominent invertebrate model species *C. elegans* and *D. melanogaster* contain rather simple regulatory networks for apoptosis compared to mammalian species (Fig. 1). This lead to the initial believe that the complexity of apoptotic mechanisms increased linearly from “simple” to “more complex” organisms. However, apoptotic mechanisms in insects and other invertebrates that parallel the complex system of mammals have been identified. This suggests that the complex apoptotic system represents the ancient condition established in early metazoans and was subsequently simplified in some species including *C. elegans* and *D. melanogaster* [5, 8, 61]. In this respect, pro-apoptotic release of cytochrome c from mitochondria as part of the endogenous apoptotic pathway has been demonstrated in mammals, sea urchins, planarians and insects, including the lepidopteran species *S. frugiperda* and *M. sexta* [18, 62–64] and few special cell types in *Drosophila* [20, 21]. Here, we describe another parallel between mammalian and insect apoptosis, namely the pro-apoptotic contribution of AChE to apoptosis of locust neurons. This finding provides further support for the existence of a complex “mammalian-like” apoptotic regulatory network in the last common ancestors of invertebrates and vertebrates.

Studies on various cell types indicated that the pro-apoptotic function of AChE is a common phenomenon in mammalian species (review: [22]). Expression of AChE increases after apoptosis induction [36] and AChE enables the assembly and activity of the apoptosome. Cytosol-located AChE has been shown to associate with cytochrome c following its release from mitochondria. This interaction is required for Apaf-1 and caspase-9 recruitment, completing apoptosome formation and initiating caspase-3 activation [31, 35]. In addition, apoptotic cells accumulate AChE in their nuclei [28, 30, 35, 36] where it acts as a nuclear DNase with similar properties as caspase-activated deoxyribonuclease (CAD) and endonuclease G [32]. Although choline-hydrolyzing activity and non-enzymatic functions of AChE may be mediated by different domains of the AChE protein (the catalytic site and a peripheral anionic region [23]) both the reduction of AChE presence and pharmacological inhibition of its catalytical function interfered with apoptotic cell death in various cell types [23, 28, 29, 31–33]. The present study may suggest that the catalytical activity of AChE promotes apoptosis in locust neurons, since hypoxia-induced cell death was prevented by NSB and TRB, two AChE inhibitors with different molecular structure, both of which have been demonstrated to inhibit insect AChE activity ( [65–68] and see below for more details). This suggests that functionality of AChE’s catalytical site is required for its pro-apoptotic role in locusts. Nonetheless, it cannot be fully excluded, that simultaneous non-catalytical interactions of locust AChE with other molecules might be altered in the presence of NSB and TRB as well.

As has been described for different cell types of various insect species, locust brain neurons also display typical characteristics of apoptotic cell death. Previous studies induced apoptosis in locust primary brain neurons by serum deprivation, H7 and hypoxia [46, 47]. Typical morphological hallmarks of apoptosis were detected in dying neurons. Most obviously and easy to quantify on the basis of fluorescent DNA labelling with DAPI [49], large and discontinuously labelled nuclei of intact cells became increasingly condensed and uniformly labelled. Chromatin condensation results from the loss of structural proteins in the nuclear matrix and represents an early event in apoptosis [69]. It typically takes place prior to DNA fragmentation by nucleases, which often leads to formation of apoptotic bodies [70, 71]. However, formation of apoptotic bodies by nucleolysis in final phases of apoptosis was only exceptionally observed in primary locust brain neurons. Instead, condensed nuclei persisted after cell membrane disintegration and loss of cytoplasm. Persistence of condensed nuclei following apoptotic cell death has previously been described in primary brain neurons of the beetle *Tribolium castaneum* [72], Schneider cells and BG2 neuronal cells from *Drosophila melanogaster* (unpublished own observations) and developmental apoptosis of *Manduca sexta* labial glands and intersegmental muscles [73]. We have previously reported the accumulation of activated caspase-3 in the cytosol of dying locust brain neurons [46]. In the present study we also observed cleaved caspase-3 immunoreactivity in nuclei that were in the process of apoptotic condensation, suggesting that activated caspase-3 translocates to the nucleus of apoptotic cells. Involvement of caspase-3 in chromatin condensation of apoptotic HeLa, Sy5y and MCF-7 cells (neuroblastoma and breast cancer respectively) has previously been described [69, 74]. Additionally, recent publications suggest that caspase-3 translocation into the nucleus may be required for chromatin condensation [75, 76]. Though TUNEL stainings successfully marked DNA double-strand breaks in nuclei of dead locust neurons, visualization of extensive DNA fragmentation (described as “DNA ladder”) in agarose gels could not be detected, despite apoptosis induction by different stressors (hypoxia, MMC, UV light). This characteristic appearance of multimers of 180–200 bp DNA fragments results from internucleosomal DNA cleavage. Conventional methods of visualizing DNA fragments failed to demonstrate internucleosomal DNA fragmentation in a variety of cell types from both mammals [77–80] and insects [73, 81, 82]. In some of these studies, more sensitive methods based on end-labeling were sufficiently sensitive to detect DNA breakdown fragments [68, 71]. Several mechanisms that may prevent the detection of DNA ladder pattern have been suggested, including involvement of different types of nucleases, contribution of caspases and AChE to DNA degradation and persistent association of histone H1 with the internucleosomal linker. Failure to detect internucleosomal DNA fragmentation may also result from insufficient sensitivity of direct DNA fragment labelling, which could be improved by selective PCR amplification of blunt 5′ phosphorylated ends [83]. TUNEL-positive staining (labels 3′ blunt ends of DNA fragments) in the nuclei of dying and dead locust primary neurons may indicate the presence of 5′ blunt ends that could be amplified by this method.

The present study investigated a potential role of AChE in apoptotic cell death of locust neurons that parallels the pro-apoptotic function of AChE in vertebrates. While vertebrates contain a single *ache* gene, that is differently spliced into three variants, most insects express two *ace* genes (*ace-1* and *ace-2)* that code for proteins with respective ACh-hydrolysing synaptic function and non-synaptic roles related to growth, reproduction and development [38, 41–43]. *Ace-1* has been identified in the European locust *L. migratoria* by sequence similarity with Oriental locust *ace-1* (*L. migratoria manilensis*). The sequence and resulting protein from Oriental locust *ace-1* showed acetylcholine hydrolysing properties and was sensitive to insecticides containing AChE inhibitors [43]. The sequence was more similar to *ace-1* from *T. castaneum*, which has been shown to mediate synaptic functions, while non-synaptic functions in this beetle were associated with *ace-2* [41]. A sequence with similarity to *Tc-ace-2* was identified at a different location in the locust genome, suggesting that locusts, as other insects, possess a second gene for AChE. Typically, *ace-2* is associated with non-synaptic functions in insects. While *ace-2* has not been studied for a potential function in insect apoptosis, *ace-1* seems to be critically involved in hypoxia-induced apoptosis of locust neurons. In order to assess, whether AChE expression changes with apoptosis-inducing stressful conditions, juvenile locusts were exposed to 6.5 or 24 h hypoxia. Our qRT-PCR data show that *Lm-ace-1* transcript increases moderately with prolonged hypoxia exposure. Since transcriptional activity might have been suppressed by the lack of ATP resulting from oxygen depletion, experiments were repeated with a reoxygenation period of 1 h in normal atmosphere between the end of hypoxia and the time of RNA isolation from brain tissues. Brains of reoxygenated animals contained slightly lower levels of *Lm-ace-1* transcript than animals that were not reoxygenated before RNA analysis. Nonetheless, our data indicate a hypoxia-dependent increase of *ace-1* expression. With reoxygenation, ATP production most probably increased, allowing the neurons to either undergo full apoptosis or recover if still possible. In the mammalian system, suppression of the presence or activity of AChE reduced, but did not fully prevent apoptosis [29, 30, 32, 35, 82]. Likewise, it seems unlikely that increased production of AChE is required for apoptosis in locust neurons, since apoptotic cells were also detected (though less frequently) in cultures supplemented with AChE inhibitors. However, promotion of apoptosome formation requires accumulation of AChE in the cytosol [31, 35] of mammalian cells. AChE is translated at the rough endoplasmic reticulum and should be targeted for export and/or association with the cell surface under normal conditions. It is currently unknown, whether a small portion of translated AChE is accidentally mislocated to the cytosol or whether a dedicated mechanism that redirects AChE to the cytosol [34] is at work here. Whether accidental mislocation or redirection to the cytosol increases with elevation of *AChE* transcript levels also remains to be demonstrated.

In this study we used two different AChE inhibitors, NSB and TRB, with different molecular structures, different binding sites and modes of interaction with AChE [84–86]. NSB is a carbamate inhibitor that covalently binds to a serine residue in the catalytic region of AChE. It has been employed in studies with several insects including cockroaches [68] and locusts [66]. A recent study demonstrated, that 100 µM, 10 µM and 1 µM NSB inhibited ~ 95%, ~ 75% and ~ 50% of AChE activity in homogenates of larval lepidoptera respectively [67]. TRB, on the other hand, is a fungal mycotoxin whose AChE inhibitory function was first characterized in a lepidopteran insect [65]. TRB establishes a very stable noncovalent binding with a larger portion of the AChE (including active site and entry of the gorge providing access to it) leading to profound conformational changes in AChE structure [84, 86]. Since 10 µM NSB and 10 µM and 1 µM TRB effectively reduced AChE activity-related staining in brain sections of *L. migratoria* (Fig. 4), we selected these concentrations, to inhibit a large portion of AChE activity in our in vitro studies with primary cultured locust brain neurons. Even though some AChE inhibitors have been demonstrated to interact with other cellular targets (e.g. other esterases and neurotransmitter receptors; reviewed in [87]), the similar effects of two different molecules on apoptotic cell death of locust brain neurons demonstrated here are likely to emerge from their common function, the inhibition of AChE’s enzymatic activity.

In order to assess a potential role of AChE in apoptotic cell death, an established in vitro approach [46, 88] with primary cultured locust brain neurons was adapted. The assay directly compares cellular survival between control and experimentally treated neuron cultures that derived from the same locust brains, minimizing variability between the treatment groups. The AChE inhibitors NSB and TRB increased the percentage of intact locust brain neurons during their cultivation for four days in normal culture conditions from 40–20% to 60–41%. Apart from the difference in the ratio of intact to dead neurons, no morphological changes between control and NSB- or TRB-treated cultures were observed. This suggested a rather specific interference of pharmacologically mediated AChE inhibition with ongoing apoptotic death in primary locust neuron cultures. In order to confirm this idea, apoptotic death was additionally stimulated by exposure of primary neuron cultures to hypoxia. Hypoxia exposure for 36 h reduced the median relative neuronal survival (compared to normoxic control cultures derived from the same locust brains) to approximately 70% (average), reproducing the efficient induction of apoptotic cell death seen in previous studies. Presence of NSB and TRB in hypoxia-treated cultures interfered with hypoxia-induced apoptosis and significantly increased neuron survival compared to hypoxia without inhibitor treatment. In fact, AChE inhibitor-treated hypoxia-exposed cultures reached the same level of neuron survival as the normoxic control cultures that derived from the same pool of brain cells. These results indicate that (in the absence of pharmacological inhibition) AChE promotes apoptosis in locust brain neurons. Whether the pro-apoptotic function of AChE is mediated through its esterase activity or other interactions with components of the apoptotic machinery cannot be decided at present state. Even though we demonstrated on brain sections that NSB and TRB are functional inhibitors of locust AChE enzymatic cleavage of choline –type substrates, locust AChE may contain other domains with distinct functions, similar to the peripheral site of vertebrate AChE. Since cytochrome c release from mitochondria has been shown to promote apoptosis in insects [19, 63] an apoptotic role for AChE in the formation and functionality of the apoptosome (similar to vertebrates) seems quite possible. Alternatively, locust AChE may promote apoptosis through cleavage of nuclear DNA (as described above for mammalian cells). Several studies reported increased expression and translocation of AChE from cytosol into the nucleus. Nonetheless (as already mentioned above), it is currently unknown why AChE appears in the cytosol, although it is translated at the endoplasmic reticulum and designated for incorporation into the cytomembrane or export from the cell.

The migratory locust has been subject to various studies on pesticide resistance and hypoxia tolerance [43, 89]. Various pesticides target AChE in order to disrupt synaptic signalling by acetylcholine, a major transmitter in insect sensory systems and central nervous neuropils. The data presented in this study indicate, that in addition to its synaptic role, locust AChE mediates an important step in neuronal apoptosis. Contribution of AChE to apoptosis regulation is another, previously undescribed mechanism shared by mammalian and insect species. Together with other functional similarities mediated by homologous molecules (see Fig. 1) in mammals, insects and other invertebrates, this provides compelling evidence for the presence of a complex regulatory network already present at the basis of metazoa [8, 62, 90]. This finding enables comparative studies that exploit specific advances of certain species to unravel apoptotic mechanisms common to many animals including humans. Our knowledge on apoptosis essentially results from studies on classical model organisms *C. elegans* and *D. melanogaster*. However, regulation of apoptosis seems to be less complex here than typically seen in other insects and vertebrates. It becomes apparent, that the genetic repertoire of *C. elegans* and *D. melanogaster* diverged more profoundly from their last common ancestor with vertebrates than that of most other invertebrates and vertebrates [91]. Since cytochrome c release from mitochondria is not required for apoptosis in most *D. melanogaster* cell types a potential contribution of AChE to the formation of the apoptosome (as described for mammalian cells) cannot be extrapolated from locust neurons but requires experiments with this species. In any case, other insects like orthopteran (locusts and crickets) and lepidopteran (silkmoths and others) species used in previous studies, may be better suited for comparative studies on the functions of AChE in apoptosis. Many degenerative diseases ultimately involve apoptotic cell death while cancer is promoted by inactivation of apoptosis. Knockdown or inactivation of AChE in normal cells decreased while induction of AChE in certain cancer cells increased their sensitivity to apoptotic stimuli [22, 24, 92]. Complete understanding of the apoptosis regulatory network will unravel new possibilities to interfere with dysregulated or disease-activated pathways. Some natural compounds [33] and synthetic molecules [23] that interfere with AChE functions have already been demonstrated to prevent apoptotic cell death. Potential use of cell-protective drugs in degenerative diseases could be explored also with support of knowledge gained from insects and other invertebrate species. We believe, that the data presented here will not only be beneficial for the understanding of apoptosis, but will also open new possibilities in the field of neuroprotection and regeneration.

### Electronic supplementary material

Below is the link to the electronic supplementary material.

Electronic supplementary material 1 Alignment of *L. migratoria* *manilensis ace-1*, *T. castaneum ace-1* and computed *L. migratoria ace-1* sequences. Coloured bases indicate dissimilarity between sequences. Identity indicates coverage amongst all three sequences. Alignment was established using Geneious Prime® (Version 2019.2.3) with implemented ClustalW (default settings) (TIF 19634 kb)

Electronic supplementary material 2 Stable expression of locust housekeeping genes. a Average raw Ct values of *Lm-18s *and *Lm-gapdh* in normoxic and hypoxic conditions. *18s rRNA* shows an average Ct of 7.21 ± 0.82 SD in normoxic conditions. Hypoxia treatment did not lead to notable shifts in expression level ( Average Ct 7.1 ± 0.72 SD). No drastic expression differences could be observed in *gapdh* expression when comparing normoxia and hypoxia (19.97 ± 7.28 SD and 20.1 ± 7.31 SD, respectively). b Average delta Ct values of housekeeping genes, normalized to normoxic controls. Both *18s rRNA* and *gapdh* expression are only slightly altered by hypoxia exposure of juvenile locusts ( 0.12 ± 0.43 and -0.13 ± 0.43, respectively). n=12 (TIF 7308 kb)

Electronic supplementary material 3 Induction of locust neuronal apoptosis by 60 µg/ml MMC a; b and UV light c; d exposure for 10 hours. a, c Anti-cleaved caspase-3 staining of stressed locust neurons. Only nuclei with beginning or completed DNA condensation display anti-cleaved caspase-3 immunoreactivity. (*) nuclei of intact neurons, (#) nuclei of dead or dying neurons. Scale bars 10 µm. b; d DNA fragmentation visualized by propidium iodide (PI) staining and TUNEL assay in locust neurons exposed to MMC (C) and UV light (D). Only nuclei with condensed PI-labelled chromatin structure (#) contain TUNEL staining while nuclei of intact neurons (*) do not. No intact cell could be localized in UV exposed cell culture. Scale bars 10 µm. e After separation of DNA from unstressed, UV-exposed and MMC-incubated locust neurons on 1.5% agarose gel no DNA fragmentation (“DNA ladder”) is detectable. 1 kb DNA ladder used as reference (TIF 11326 kb)

## Data Availability

Locust *ace-1* sequence was predicted by alignment of *Locusta migratoria manilensis ace-1* (Accession number: EU231603) with 99% coverage (e-value 0) and *Tribolium castaneum ace-1* (Accession number: HQ260968) with 73% coverage (e-value 4e-56) against locust genome available on i5k platform (https://i5k.nal.usda.gov/locusta-migratoria). All raw data can be accessed on request.

## Appendix

### Lm-ace1 Sequence (560 bp)

TGCCCATTCG CAGTTTGATG TCATACATTC TGTGAATATG AAATAATAAC AACTGCAGGATCAGAAGTGA TGTCTGTTAT TTCGGAATAG ACGCCACATG TGTATATTCT TTACTCCTCCTTTTGAAATG GCGGTGGTAG CGCTAGGGCT GAACGGCCGT CCTGCCCGGC AGGTGTTCGGCGACTTCCCG GGCTCGGTGA TCTGGAACCC GAACACGCAG CTGTCGGAGG ACTGCCTGTACATCAACGTG GTGGCGCCCA AGCCGCGGCC GCGCAACGCC GCCGTCATGG TGTGGATCTTCGGCGGCGGC TTCTACTCGG GCACGGCGAC GCTCGACGTG TACGACCACA AGACGCTGGTGTCCGAGGAG AACGTGATCC TGGTGTCGAT GCAGTACCGC GTCGCCTCGC TCGGCTTCCTCTTCTTCGAC ACGAGCGACG TGCCGGGCAA CGCGGGGCTC TTCGACCAGC TGATGGCGCTGCAGTGGGTG CACGACAACA TCCACTACTT CGGCGGAAAC CCGCACAACG TGACGCTGTTCGGCGAGTCG GCGGGCGCCG.

**Table 3:** Blast hits of *L. migratoria manilensis ace-1* and *T. castaneum ace* sequences on *L. migratoria* genome

	*L. migratoria manilensis* (EU231603)	*T. castaneum ace-1* (HQ260968)	*T. castaneum ace-2 (HQ260969)*
Scaffold Hit	28,704	28,704	142,838
Coverage	99.50%	73%	75.70%
Sequence length	620 bp	386 bp	342 bp
Mismatches	3	104	67
Start (Sequence)	251.798	251.566	344.928
End (Sequence)	251.179	251.206	345.254
E Value	0	4E-56	1E-56

Blast was run on i5k platform

**Table 4:** Coverage [%] of reference sequences to predicted *L. migratoria ace* sequence

	*L. migratoria manilensis ace-1*	*L.migratoria ace* Consensus sequence	*T. castaneum ace-1*	*T. castaneum ace-2*
*L. migratoria manilensis ace-1*		99.5	59.1	45.5
*L.migratoria ace*	99.5		60.3	46.7
*T. castaneum ace-1*	59.1	60.3		45.5
*T. castaneum ace-2*	45.5	46.7	45.5	

**Table 5:** Ct values of locust housekeeping genes in normoxic and hypoxic conditions

Biological Replicates	Normoxia *Lm-18 s rRNA*		Hypoxia *Lm-18 s rRNA*		Normoxia *gapdh*		Hypoxia *gapdh*	
	Average Ct	∆Ct	Average Ct	∆Ct	Average Ct	∆Ct	Average Ct	∆Ct
1	9.08	0	9.07	0.02	18.95	0	19.51	−0.57
2	7.58	0	6.95	0.63	18.95	0	19.51	−0.57
3	7.30	0	7.39	−0.09	21.07	0	21.08	−0.01
4	6.41	0	7.10	−0.69	20.20	0	20.71	−0.51
5	6.78	0	6.77	0.01	20.18	0	20.16	0.03
6	6.78	0	6.77	0.01	19.67	0	19.95	−0.28
7	8.55	0	7.70	0.86	19.67	0	19.95	−0.28
8	6.76	0	6.59	0.16	20.69	0	20.06	0.63
9	6.97	0	6.99	−0.01	20.69	0	20.06	0.63
10	6.42	0	6.77	-0.35	20.20	0	20.20	0.00
11	6.99	0	6.79	0.20	19.67	0	20.42	−0.75
12	6.91	0	6.29	0.62	19.68	0	19.61	0.07
Average	7.21	0	7.10	0.12	19.97	0	20.10	−0.13
STDV	± 0.82	± 0	± 0.72	± 0.43	± 7.28	± 0	± 7.31	± 0.43

Expression of *Lm-gapdh* and *Lm-18 s* *rRNA* as housekeeping genes in hypoxic conditions
