# Supplementary figures and images for: Acetylcholinesterase promotes apoptosis in insect neurons
Source: Apoptosis. 2020 Aug 5;25(9):730–46. doi: 10.1007/s10495-020-01630-4 (PMC7527371; doi:10.1007/s10495-020-01630-4)

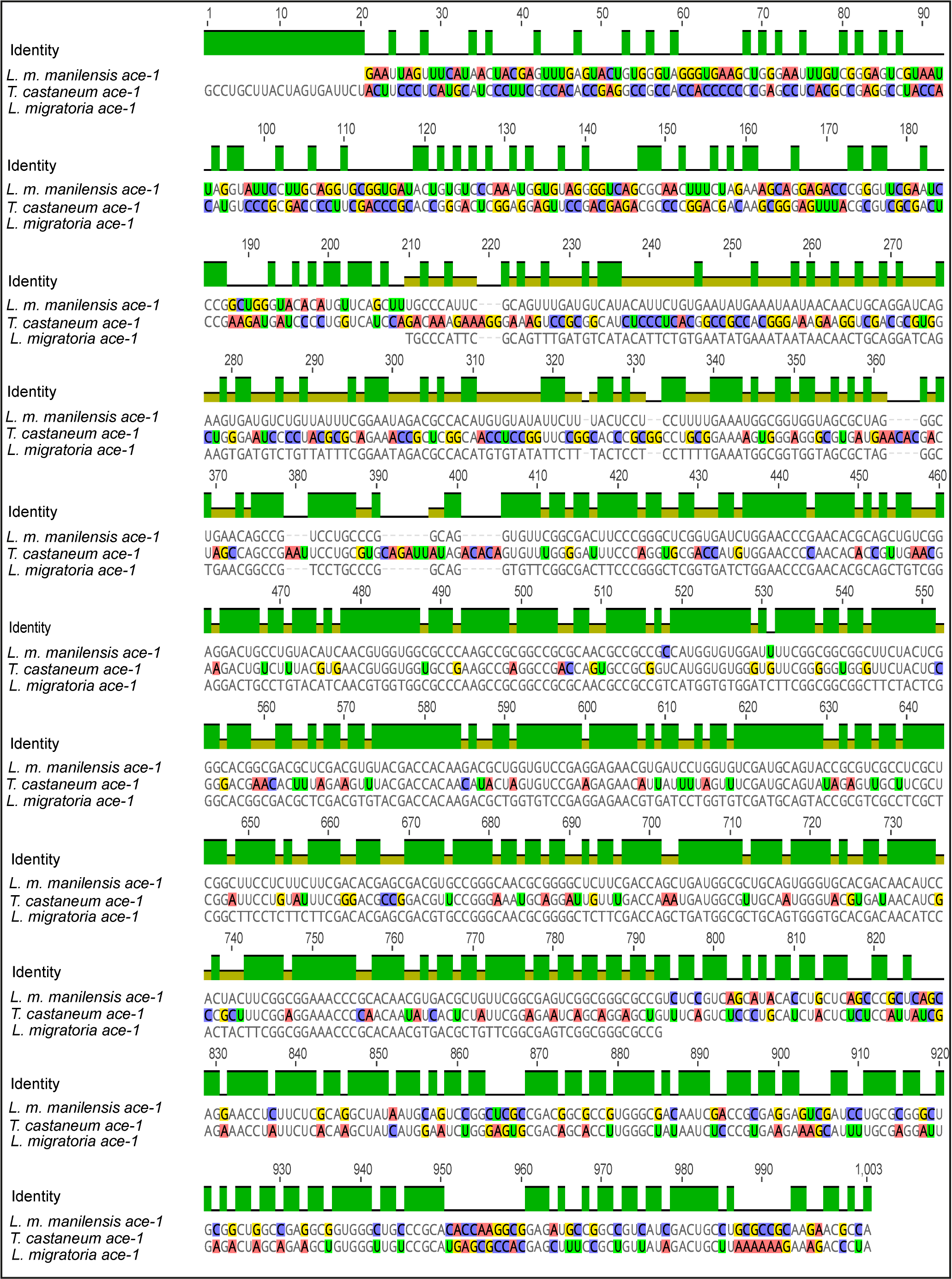

Supplement: Supplementary file 1 — Electronic supplementary material 1 Alignment of L. migratoria manilensis ace-1, T. castaneum ace-1 and computed L. migratoria ace-1 sequences. Coloured bases indicate dissimilarity between sequences. Identity indicates coverage amongst all three sequences. Alignment was established using Geneious Prime® (Version 2019.2.3) with implemented ClustalW (default settings) (TIF 19634 kb) [file 10495_2020_1630_MOESM1_ESM.tif]

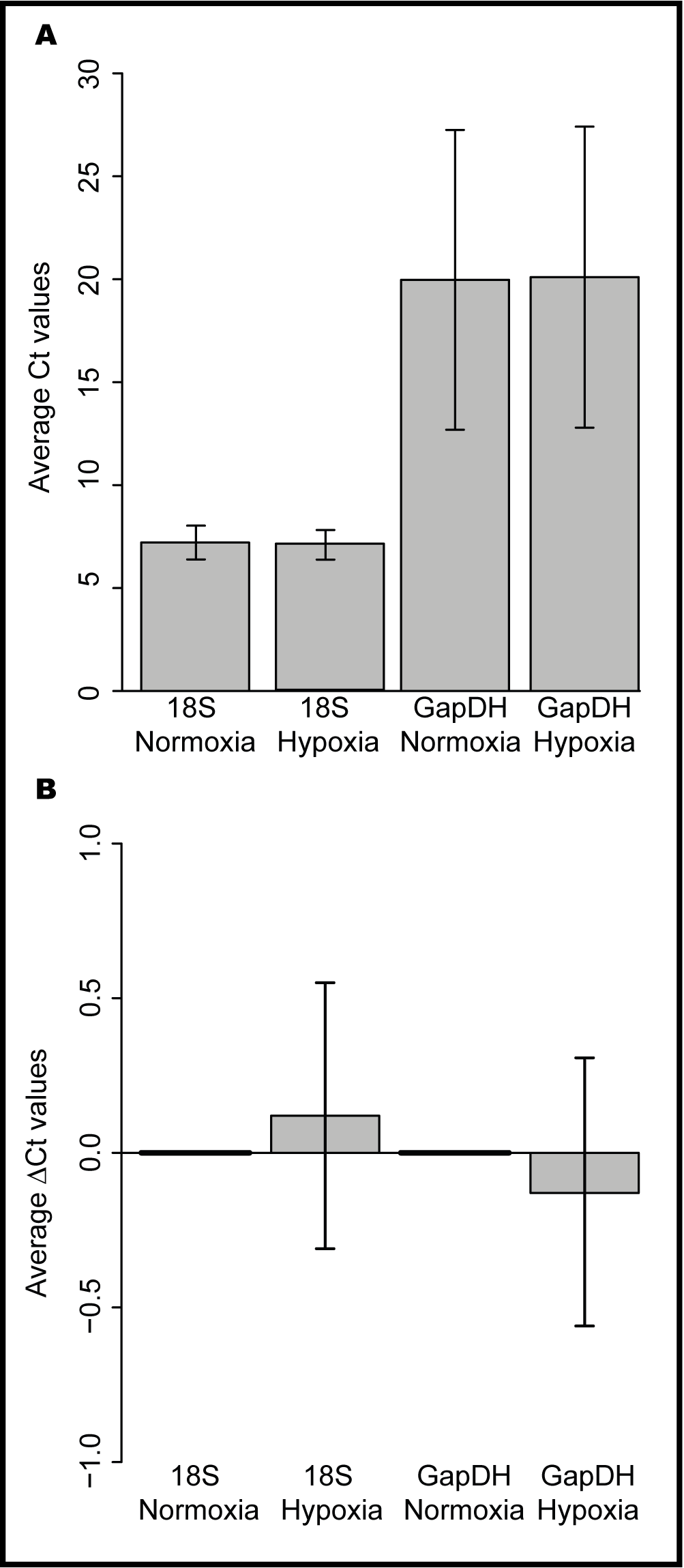

Supplement: Supplementary file 2 — Electronic supplementary material 2 Stable expression of locust housekeeping genes. a Average raw Ct values of Lm-18s and Lm-gapdh in normoxic and hypoxic conditions. 18s rRNA shows an average Ct of 7.21 ± 0.82 SD in normoxic conditions. Hypoxia treatment did not lead to notable shifts in expression level ( Average Ct 7.1 ± 0.72 SD). No drastic expression differences could be observed in gapdh expression when comparing normoxia and hypoxia (19.97 ± 7.28 SD and 20.1 ± 7.31 SD, respectively). b Average delta Ct values of housekeeping genes, normalized to normoxic controls. Both 18s rRNA and gapdh expression are only slightly altered by hypoxia exposure of juvenile locusts ( 0.12 ± 0.43 and -0.13 ± 0.43, respectively). n=12 (TIF 7308 kb) [file 10495_2020_1630_MOESM2_ESM.tif]

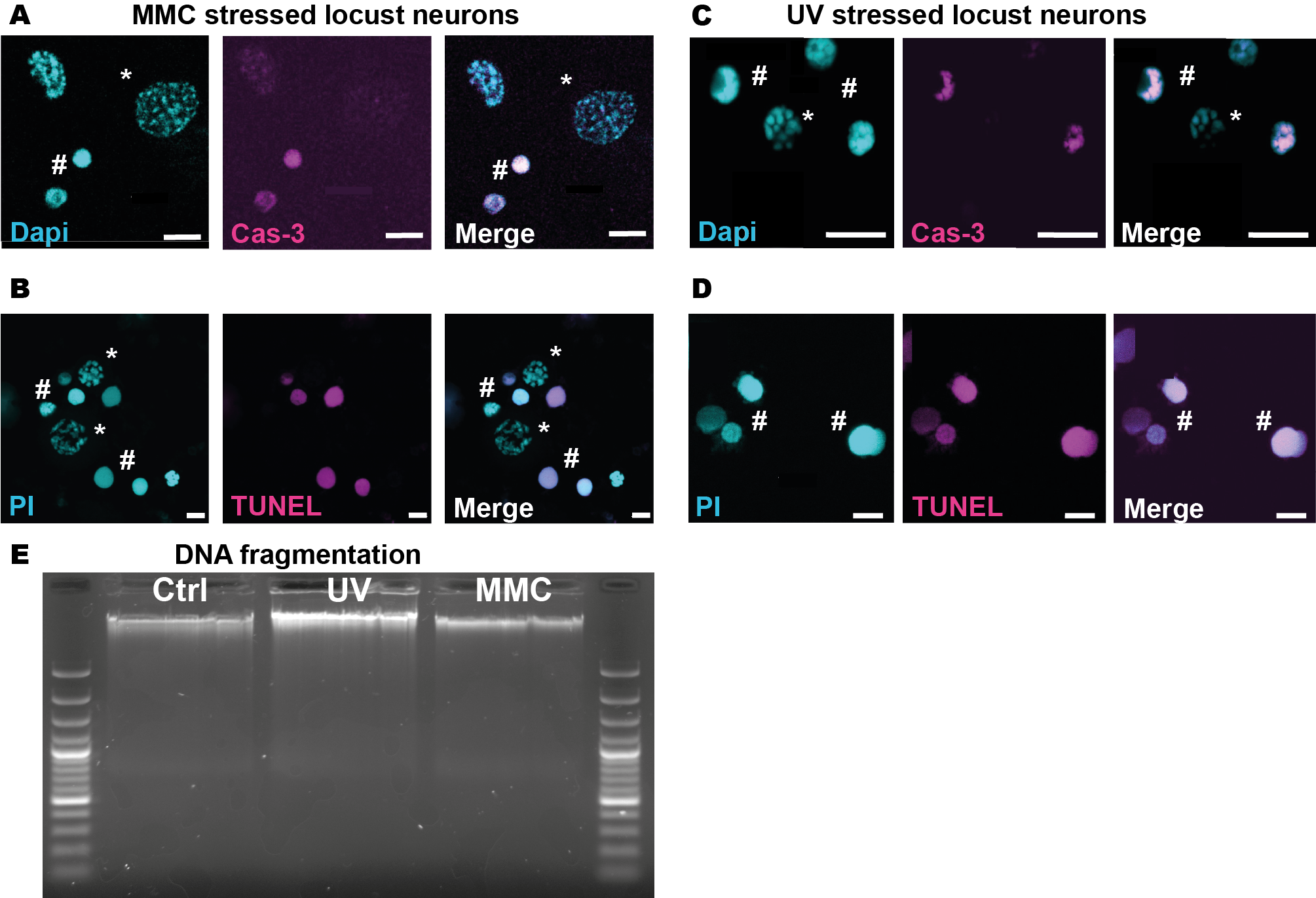

Supplement: Supplementary file 3 — Electronic supplementary material 3 Induction of locust neuronal apoptosis by 60 µg/ml MMC a; b and UV light c; d exposure for 10 hours. a, c Anti-cleaved caspase-3 staining of stressed locust neurons. Only nuclei with beginning or completed DNA condensation display anti-cleaved caspase-3 immunoreactivity. (*) nuclei of intact neurons, (#) nuclei of dead or dying neurons. Scale bars 10 µm. b; d DNA fragmentation visualized by propidium iodide (PI) staining and TUNEL assay in locust neurons exposed to MMC (C) and UV light (D). Only nuclei with condensed PI-labelled chromatin structure (#) contain TUNEL staining while nuclei of intact neurons (*) do not. No intact cell could be localized in UV exposed cell culture. Scale bars 10 µm. e After separation of DNA from unstressed, UV-exposed and MMC-incubated locust neurons on 1.5% agarose gel no DNA fragmentation (“DNA ladder”) is detectable. 1 kb DNA ladder used as reference (TIF 11326 kb) [file 10495_2020_1630_MOESM3_ESM.tif]
